# Acromioplasty reduces critical shoulder angle in patients with rotator cuff tear

**DOI:** 10.1371/journal.pone.0253282

**Published:** 2021-06-30

**Authors:** Che-Li Lin, Li-Fong Lin, Tzu-Herng Hsu, Lien-Chieh Lin, Chueh-Ho Lin, Shih-Wei Huang

**Affiliations:** 1 Department of Orthopedic Surgery, Shuang Ho Hospital, Taipei Medical University, New Taipei City, Taiwan; 2 Department of Orthopedics, School of Medicine, College of Medicine, Taipei Medical University, Taipei, Taiwan; 3 Department of Physical Medicine and Rehabilitation, Shuang Ho Hospital, Taipei Medical University, Taipei, Taiwan; 4 School of Gerontology Health Management, College of Nursing, Taipei Medical University, Taipei, Taiwan; 5 Master Program in Long-Term Care, College of Nursing, Taipei Medical University, Taipei, Taiwan, R.O.C; 6 Center for Nursing and Healthcare Research in Clinical Practice Application, Wan Fang Hospital, Taipei Medical University, Taipei, Taiwan, R.O.C; 7 Department of Physical Medicine and Rehabilitation, School of Medicine, College of Medicine, Taipei Medical University, Taipei, Taiwan; North Carolina State University, UNITED STATES

## Abstract

Critical shoulder angle (CSA) is the angle between the superior and inferior bone margins of the glenoid and the most lateral border of the acromion and is potentially affected during a rotator cuff tear (RCT). Acromioplasty is generally performed to rectify the anatomy of the acromion during RCT repair surgery. However, limited information is available regarding the changes in the CSA after anterolateral acromioplasty. We hypothesized that CSA can be decreased after anterolateral acromioplasty. Data were retrospectively collected from 712 patients with RCTs and underwent arthroscopic rotator cuff repair between January 2012 and December 2018, of which 337 patients were included in the study. The presurgical and postsurgical CSA were then determined and compared using a paired samples *t* test. Because previous study mentioned CSA more than 38 degrees were at risk of rotator cuff re-tear, patients were segregated into two groups: CSA < 38° and CSA ≥ 38°; these groups were compared using an independent-samples *t* test. These 337 participants (160 male and 177 female) presented a CSA of 38.4° ± 6.0° before anterolateral acromioplasty, which significantly decreased to 35.8° ± 5.9° after surgery (*P* < .05). Before surgery, 172 patients were present in the CSA ≥ 38° group and 57 were preset in the CSA < 38° group after surgery. The CSA decreased significantly in the CSA ≥ 38° group rather than in the CSA < 38° group (*P* < .05). In conclusion, the CSA can be effectively decreased through anterolateral acromioplasty, and this reduction in the CSA is more significant among individuals with CSA ≥ 38° than among those with CSA < 38°, indicating that acromioplasty is recommended along with RCT repair especially among individuals with a wide presurgical CSA.

## Introduction

Rotator cuff tears (RCTs) are one of the most common shoulder girdle disorders, requiring surgical repair. RCT is a degenerative rotator cuff disorder and is expected to be highly prevalent among older individuals [1, 2]. Among the individuals aged >60 years, the prevalence of RCTs is estimated as >10% [3]. RCTs often result in functional deterioration and increase the economic health care burden [4]. RCT pathogenesis remains somewhat unclear. RCTs result from numerous factors, which are classified as intrinsic, extrinsic, or both [5]. Chronic overload on rotator cuff tendons is considered a contributor to RCTs [6]. The correlation between RCT prevalence and scapular geometric factors including the acromial index and the critical shoulder angle (CSA) have recently received increasing attention as potential risk factors of RCTs [7–9].

The CSA was mentioned since 2013 and refers to the angle between the superior and inferior bony margin of the glenoid and the lateral margin of the acromion [10] and indicates glenoid inclination and lateral acromion extension. Moor et al reported that at CSA > 35°, patients had an increased risk of rotator cuff injury [11]. Moreover, patients with an RCT with CSA > 38° presented a higher retear rate after RCT repair surgery [12, 13]. Furthermore, Gerber et al reported that patients with RCTs after rotator cuff repair surgery with CSA > 35° had a higher retear rate and unfavorable outcomes than those with CSA < 33° after acromioplasty [14]. The CSA can be easily quantified and evaluated through standard radiographic imaging of the shoulder.

Acromioplasty, is performed to modify existing acromial architecture to eliminate mechanical impingement and has emerged as one of the most frequently performed orthopedic surgical procedures. Lateral acromioplasty can directly reduce the CSA [12, 15, 16], as can anterior acromioplasty [17]. However, limited information is available regarding the changes in the CSA among Asian individuals after acromioplasty, through a large cohort study. Therefore, this study aimed to evaluate changes in the CSA after acromioplasty among Asian individuals undergoing RCT repair surgery and investigate the factors contributing to postsurgical retear among them. We hypothesized that acromioplasty can reduce the CSA of RCT repair patients. For clinical implication, decrease the CSA especially for those with high CSA patients could lessen the risk of further re-tear and surgery. Our study could provide the data of CSA reduction for clinical application.

## Methods

### Study design and participants

In this retrospective study, patients with RCTs undergoing arthroscopic repair surgery via anterolateral acromioplasty in a medical university hospital from January 2012 to December 2018 were recruited. All the participants were recruited from the orthopedic department, and this study was approved by the institutional review board of the medical university (IRB N201707024). The data were deidentified, and the requirement for informed consent was waived owing to the retrospective nature of study. All eligible patients were retrospectively assessed through their medical charts in accordance with the inclusion and exclusion criteria. The inclusion criteria were as follows: (1) age > 20 years; (2) unilateral, degenerative, full-thickness RCT diagnosed through magnetic resonance (MR) arthrography and corroborated during arthroscopic rotator cuff repair surgery with anterolateral acromioplasty; and (3) data available regarding shoulder radiographic imaging before and after within 6 months of receiving anterolateral acromioplasty. The exclusion criteria were (1) history of shoulder surgery, (2) glenohumeral osteoarthritis or acromioclavicular arthritis (which could influence CSA measurements), and (3) shoulder radiographic image quality too poor for assessment. Demographic data including age, sex, body mass index (BMI), affected side, hyperlipidemia, and diabetes mellitus were recorded.

### Standardized surgical technique

All participants underwent a standardized surgical procedure by two orthopedic surgeons specialized in arthroscopic surgery. A posterior arthroscopic portal was used to assess the intraarticular structure of the shoulder to diagnose full-thickness RCTs. Using an anterior portal, the border of the anatomical structure was cleaned using a full-radius resector. Biceps tenotomy without tenodesis and medial release of the coracohumeral and superior glenohumeral ligaments were performed as previously reported [18, 19]. Through the anterolateral portal, bursectomy was performed using a shaver. Anterolateral acromioplasty was performed using the posterior portal of an arthroscope with a shaver blade designed to cut soft tissue or bones, and the acromion appeared distinct from the soft tissue and lateral boarder of the acromion. Thereafter, the shaver blade was used to resect the most lateral boarder of the acromion in the anteroposterior and inferosuperior directions [15].

### Radiographic assessment of CSA

**Fig 1:**
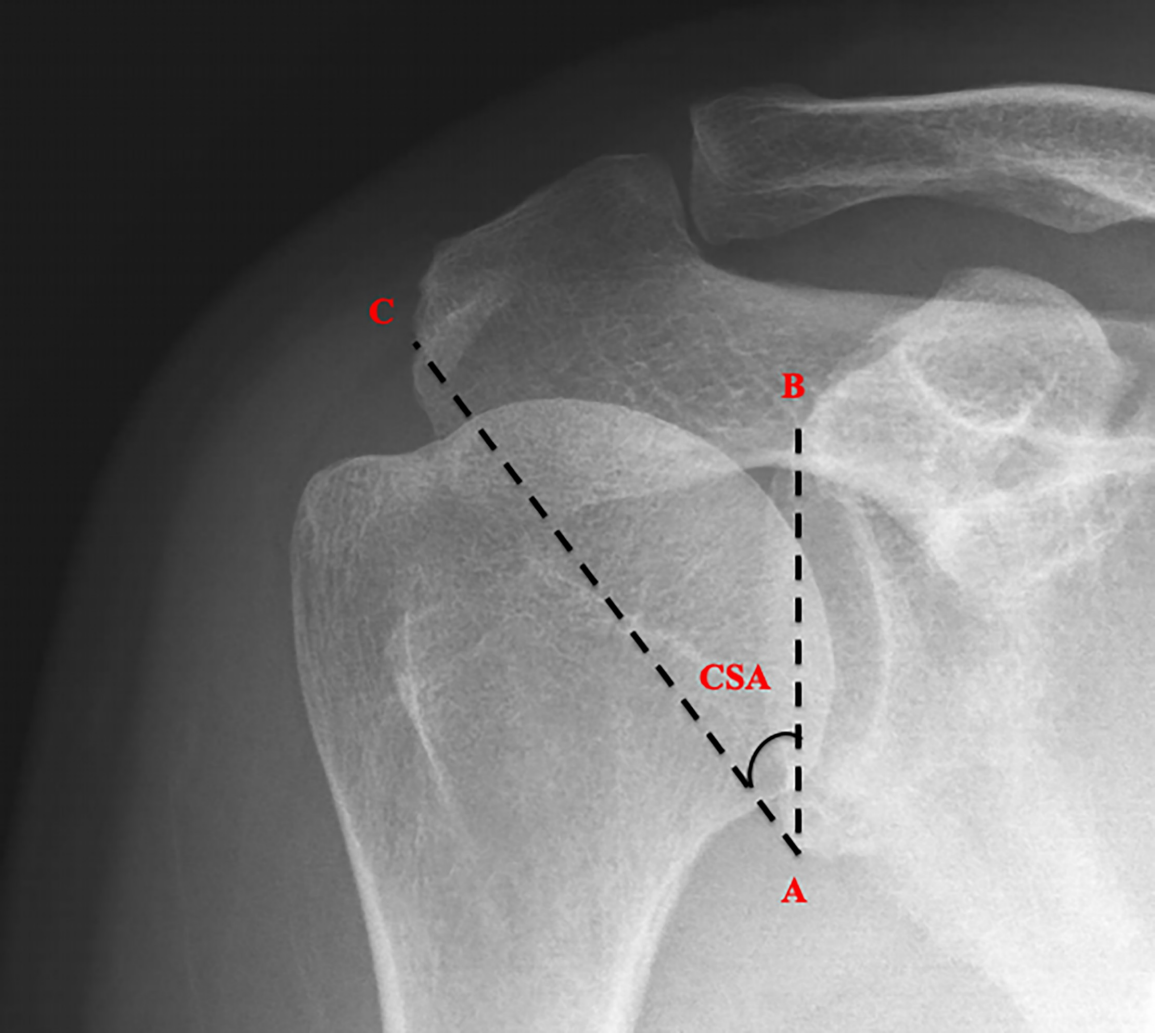
Critical shoulder angle formed by a line joining the inferior (point A) and superior (point B) border of the glenoid fossa and another line joining the inferior border of the glenoid (point A) with the inferior lateral boarder of the acromion (point C).

Conventional anterior–posterior shoulder radiography was performed before and after surgery. Throughout the evaluation period, the preoperative radiographic images were obtained <3 months before surgery and postoperative images were obtained<3 months after surgery, using a standardized protocol. These images were obtained using an upright standing posture and a descending beam tilted to 20° to ensure accuracy of the CSA assessment. We used a CSA measurement protocol reported by Blonna et al. [20] To prevent the effects of scapular rotation and beam projection angle difference, we adopted standardized measurement protocols and imaged the shoulder in the true anterior–posterior view with a digitally embedded tool. Providing the radiographic images did not overlap and during rotation along the edges of the glenoid cavity, image quality remained sufficient to evaluate these parameters. Fig 1 summarizes the CSA measurements. The angle was measured from a line connecting superior and inferior bone margins of the glenoid and a line from the inferior bony margin of the glenoid to the most lateral border of the acromion. Regarding the accuracy of these parameter measurements, two independent evaluators blinded to the presurgical and postsurgical imaging findings measured the CSA among all participants using standardized evaluation methods with repeated inter-session and intra-session measurements. Thereafter, the radiographic images were randomly evaluated thrice per image, thus yielding six values in total. The mean value of these values was obtained for data analysis. Based on a previous study, the inter- and intra-observer reliability for measuring the CSA was excellent, with an intra-class coefficient (ICC) more than 0.9 [21].

### Statistical analysis

Categorical variables are presented as percentages, and continuous variables as means ± standard deviations. We conducted a paired samples *t* test for comparing the presurgical and postsurgical CSA values. Based on a previous study, reporting a risk of RCTs at CSA > 38°, we subdivided the all the patients into two groups (CSA ≥ 38° and CSA < 38°) to evaluate the effect of anterolateral acromioplasty among different groups [22]. Changes in the CSA were evaluated using ANOVA analysis. Furthermore, the proportion of individuals in the CSA < 38° group after surgery were compared using a chi-square analysis. All statistical analyses were performed using the Statistical Package for Social Sciences (version 19.0), and *P* < .05 was considered statistically significant.

## Results

**Fig 2:**
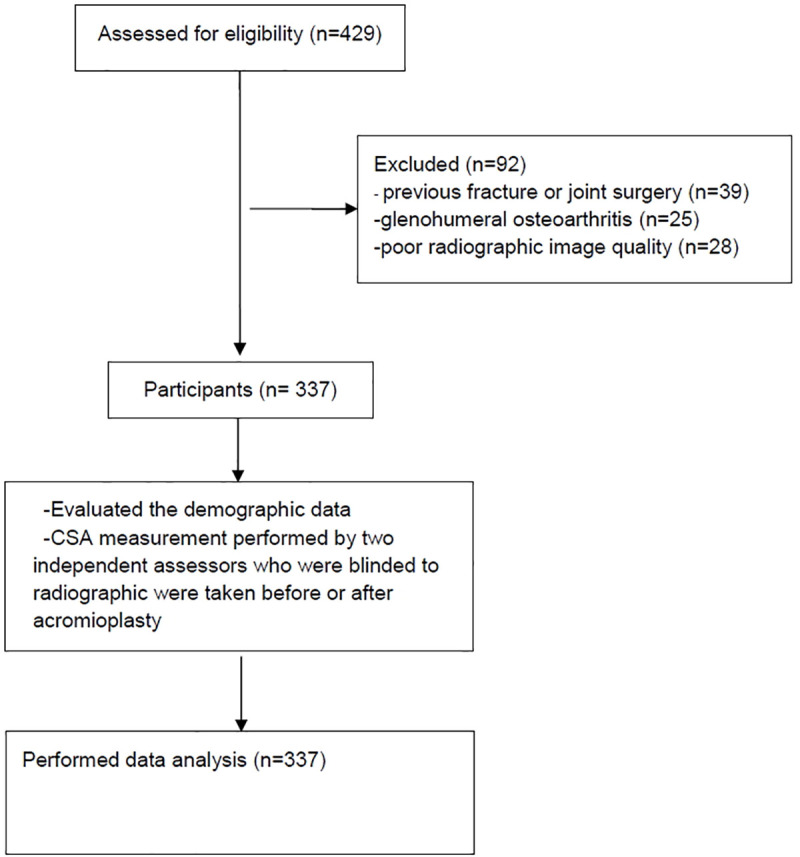
Schematic of the study protocol.

**Fig 3:**
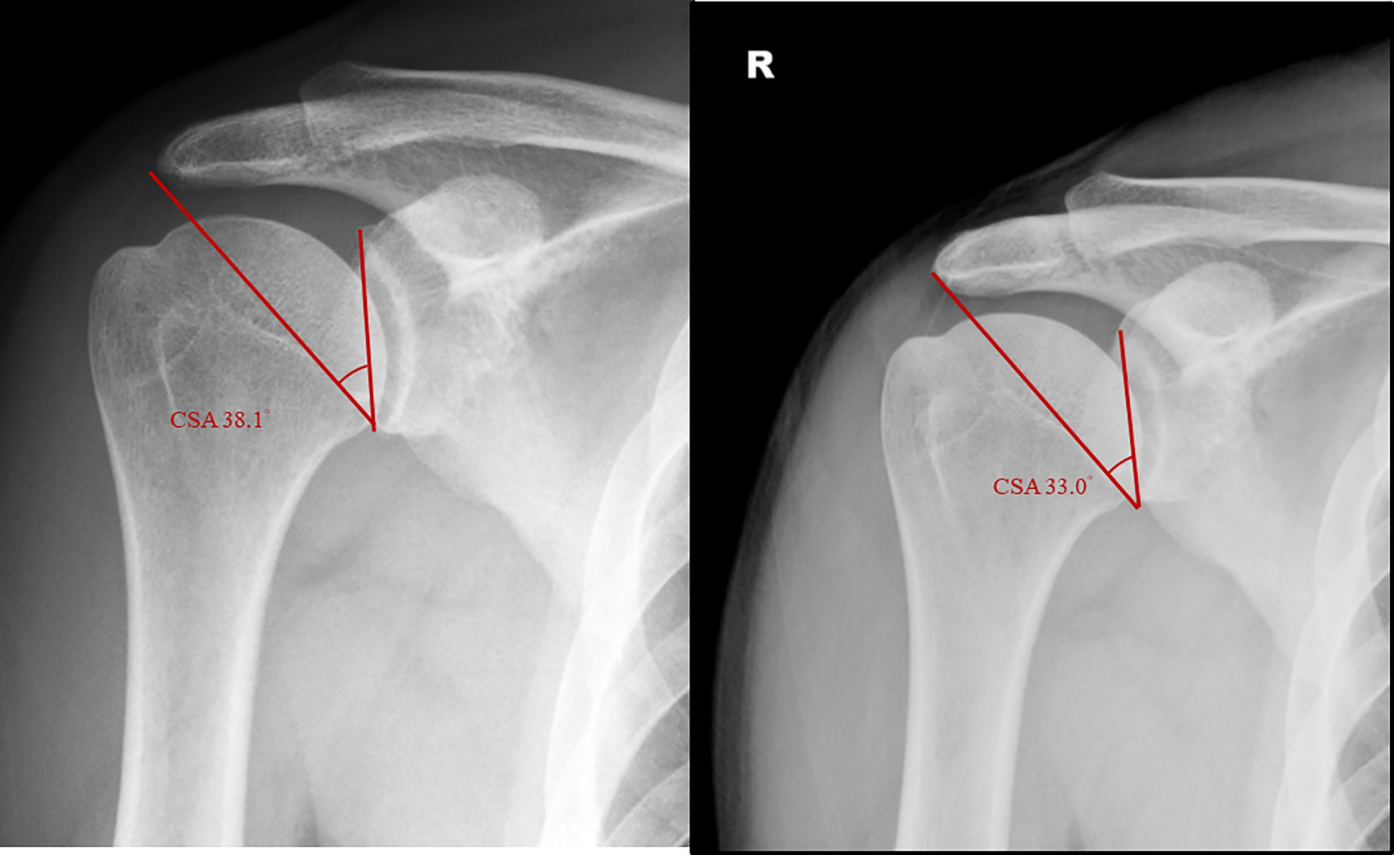
Demonstration of critical shoulder angle image changes before and after anterolateral acromioplasty.

**Fig 4:**
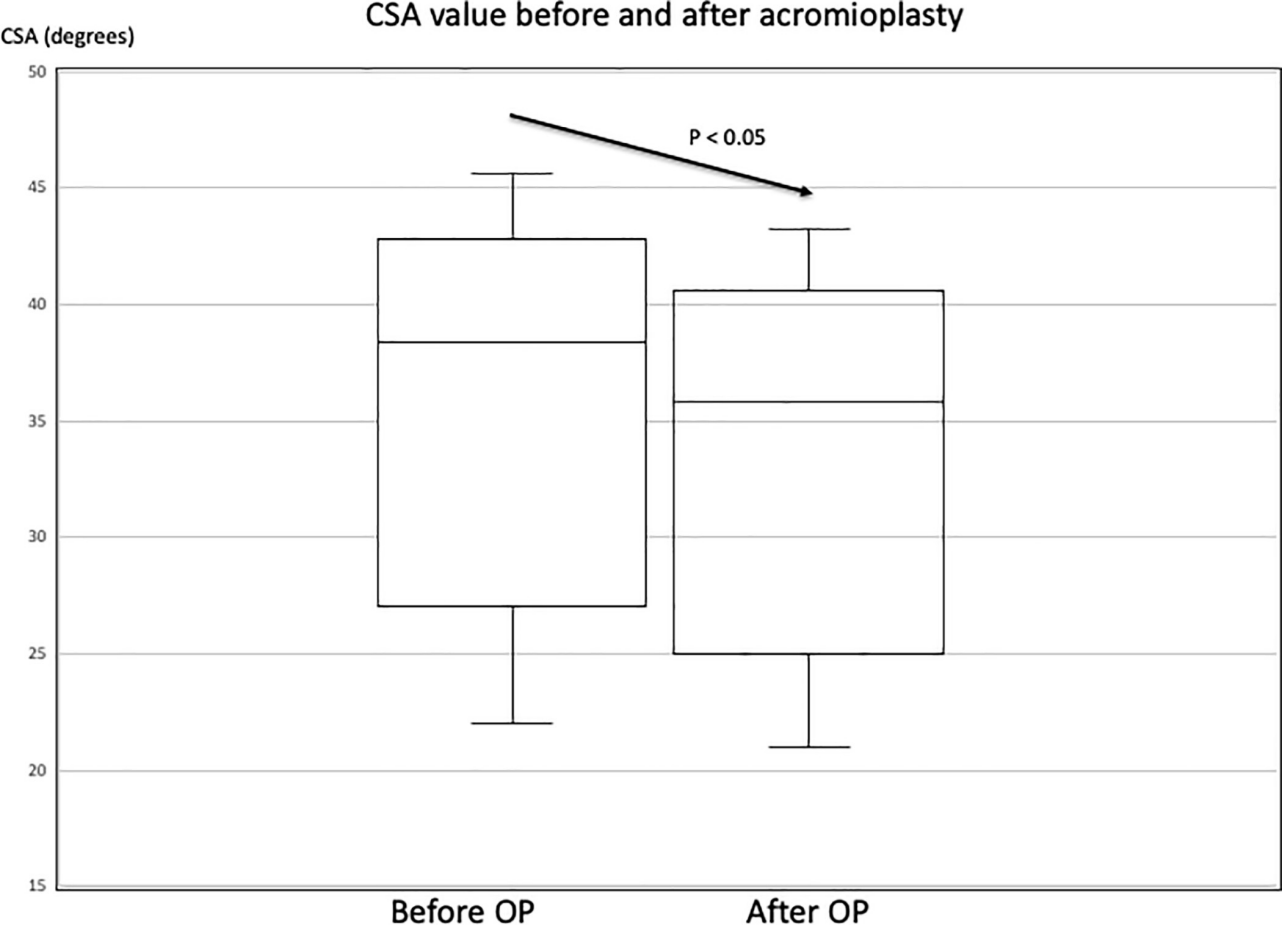
Critical shoulder angle before and after anterolateral acromioplasty.

In total, 337 of 712 patients fulfilled the inclusion and exclusion criteria. A graphical representation of the study protocol is provided in Fig 2. In our study, the intrarater and interrater accuracy for CSA was 95% and 91%, respectively. The demographic data of the patients are listed in Table 1. The Fig 3 demonstrated the CSA before and after acromioplasty of one of these RCT repair patients. The average presurgical CSA was 38.4° ± 6.0° and significantly decreased to 35.8° ± 5.9° after acromioplasty (*P* < .05; Fig 4); the average reduction in the CSA being 2.6° among all patients. Among patients with CSA ≥ 38°, anterolateral acromioplasty significantly reduced the CSA by 4.14° on average (*P* < .05), while that among patients with CSA < 38° was nonsignificant at 1.02°. (Table 2) Before acromioplasty, 172 patients had CSA ≥ 38°, and this number significantly decreased to 114 after the procedure.

**Table 1:** Demographic characteristics of the patients undergoing anterolateral acromioplasty (n = 337).

Variables	
Age, y	64.2 ± 10.1
Sex, n (male)	160
Surgery on dominant side, n	215
BMI, kg/m^2^	25.5 ± 3.7
Type 2 diabetes mellitus, n	83
Hyperlipidemia, n	30

Continuous data are presented as means ± standard deviations, and categorical data as numbers of patients.

**Table 2:** Critical shoulder angle (CSA) changes after acromioplasty among all the participants and subgrouping by CSA of 38 degrees.

CSA	All	CSA≥38°	CSA<38°	P value^a^
	n = 337	n = 172	n = 165	
Before acromioplasty	38.4° ± 6.0°	43.2°±3.9°	33.4°±3.0°	
After acromioplasty	35.8° ± 5.9°	39.1°±5.2°	32.4°±4.5°	
Changes	2.6°±4.3°^b^	4.1°±4.1°^b^	1.0°±3.9°	<0.001

^a^P value was calculated by ANOVA with comparing CSA≥38° and CSA<38° groups

^b^Paired t test with comparing before and after acromioplasty, P < 0.05.

## Discussion

We purpose the CSA, which is related to the risk of rotator cuff re-tear and further surgery, can be reduced by acromioplasty. This study reports that anterolateral acromioplasty can reduce the CSA among patients undergoing arthroscopic RCT repair surgery. Among individuals with CSA ≥ 38°, acromioplasty can bring about a 33% reduction in the CSA to <38°, which has been previously reported as a risk predictor of RCTs. Moor et al reported that the CSA reflects the coverage of the acromion, including the glenoid inclination, and a larger CSA was associated with RCT. Moor et al compared an RCT group with a normal group and reported that their CSAs were 38° and 33.1°, respectively, and the threshold point was 35°. A previous study investigated the potential of the CSA for predicting RCT in Asian individuals experiencing shoulder pain and reported a threshold of 38° [23]. Our study findings indicated that as long as RCT patients with CSA more than 38°, acromioplasty is recommended during the repair surgery for effective reduce the CSA, which is related with subsequent re-tear incidence.

Lateral acromioplasty is considered to reduce the load of the supraspinatus and increase the activity of the deltoid muscles during scapular-plane elevation, which can benefit supraspinatus tendons with a potentially lower chronic overload on subsequent injury. Although anterior acromioplasty is not a mechanical and experimental rationale for reducing the CSA through lateral acromion resection, a recent study reported that this method can reduce the CSA after surgery [17]. Moreover, another study reported that anterior acromioplasty can relieve shoulder pain [24]. However, another previous study reported that only anterior acromioplasty cannot prevent RCTs [25]. Therefore, we performed anterolateral acromioplasty herein.

A biomechanical study reported that a large CSA can increase the shear forces in the shoulder joint and thus reduce the stability of the shoulder joint, indicating the association between RCTs and CSA [26]. Hence, surgical correction of the large CSA through acromioplasty can reduce the retear rate after RCTs repair surgery [20, 27]. A cadaveric study reported that arthroscopic lateral acromioplasty can reduce the CSA by 1.4° ± 0.6° [15]. The present study reports more obvious changes in the CSA with an average reduction of 2.6°. Among the present patients with RCTs with CSA ≥ 38°, the CSA could be reduced in 57 patients within the previously reported threshold value [23]. Anterolateral acromioplasty can help achieve a safer CSA among individuals with RCTs to reduce the postsurgical retear risk.

The quantitative effects of acromioplasty on the CSA have remained unclear thus far. Katthagen et al reported a CSA of 2.8° ± 0.7° after 5-mm lateral acromioplasty among cadavers [15]. Gerber et al assessed 49 patients and reported a CSA reduction of 3.6° after 6-mm lateral acromioplasty [14]. Another cadaveric study by Altintas et al reported that 10-mm lateral acromioplasty can reduce the CSA by approximately 10° [28]. The present results are concurrent with those of Katthagen et al with regards to CSA reduction. Furthermore, individuals with a higher CSA presented favorable outcomes on acromioplasty and achieved a safer CSA. In clinical practice, the border of the lateral acromion may greatly protrude and more regions can be assessed for resection. However, the present retrospective study could not accurately determine the distance of the acromion resected among individuals with RCTs undergoing acromioplasty. The quantitative effect of the extent of lateral acromial resection on the reduction in the CSA should be assessed further.

Although this study reports a mean CSA of 35.8° after acromioplasty, which was lesser than that reported previously, 114 individuals still had CSA ≥ 38°. Interindividual differences in acromial anatomy may have resulted in the reduction in the CSA even on using the same anterolateral resection technique among these patients. Despite standardized surgical procedures, the precise value could not be determined among individuals undergoing lateral acromioplasty, thus also potentially leading to differences in the changes in the CSA among the present patients. Moreover, the definite value for anterolateral resection of the acromion was not evaluated before surgery. Gerber et al reported a method to determine the extent of the resection through true anteroposterior radiography and equated this value to the undersurface distance of the target acromion, using a probe with a caliber and via electrocautery [14]. Furthermore, they performed MR imaging to determine the angle between the lateral boarder of the acromion and the glenoid fossa to precisely correct the CSA [14]. Additional studies on the application of this method are required to determine the accuracy and efficacy among individuals with RCTs undergoing acromioplasty.

This study reports the effect of lateral acromioplasty in decreasing the CSA, which is a risk factor for RCT. Nevertheless, several limitations should be addressed herein. First, despite a good interrater and intrarater accuracy of measurements, the measurement bias should be considered herein. For more accurate measurement, we randomly assigned the standardized anterior–posterior shoulder radiographic images to two independent observers, who were unaware these images were obtained before or after acromioplasty. Furthermore, by preventing the bias through radiographic imaging, we attempted to standardize the protocol and exclude low-quality images, thus potentially influencing the reliability of measurements. Second, retrospective design of the study served as a limitation. To prevent heterogeneous data collection, we only selected two senior orthopedic surgeons through the same surgical method with the same training background. Third, the extent of acromion resection was not evaluated in this retrospective study. The optimal amount of resection could not be defined for CSA correction herein. Finally, all participants had the same racial background, namely East Asian, and racial differences may result in differences in the CSA anatomy.

## Conclusion

Anterolateral acromioplasty can reduce the CSA during RCT repair surgery. In particular, among individuals with CSA ≥ 38°, being at the risk of subsequent rotator cuff retear, acromioplasty could effectively reduce the CSA by 4.14° on average among these patients. This study provides data regarding the changes in the CSA after anterolateral acromioplasty among East Asian individuals. Additional prospective cohort studies are required to investigate the prognosis after RCT repair with or without acromioplasty.

## Supporting information

S1 Data (XLS)

## References

[1] Nakagawa Y, Hyakuna K, Otani S, Hashitani M, Nakamura T. Epidemiologic study of glenohumeral osteoarthritis with plain radiography. Journal of shoulder and elbow surgery. 1999;8(6):580–4. Epub 2000/01/14. doi: 10.1016/s1058-2746(99)90093-9 . 10633892

[2] Minagawa H, Yamamoto N, Abe H, Fukuda M, Seki N, Kikuchi K, et al. Prevalence of symptomatic and asymptomatic rotator cuff tears in the general population: From mass-screening in one village. J Orthop. 2013;10(1):8–12. Epub 2014/01/10. doi: 10.1016/j.jor.2013.01.008 ; PubMed Central PMCID: PMC3768248. 24403741 PMC3768248

[3] Reilly P, Macleod I, Macfarlane R, Windley J, Emery RJ. Dead men and radiologists don’t lie: a review of cadaveric and radiological studies of rotator cuff tear prevalence. Ann R Coll Surg Engl. 2006;88(2):116–21. Epub 2006/03/23. doi: 10.1308/003588406X94968 ; PubMed Central PMCID: PMC1964063. 16551396 PMC1964063

[4] Paloneva J, Koskela S, Kautiainen H, Vanhala M, Kiviranta I. Consumption of medical resources and outcome of shoulder disorders in primary health care consulters. BMC Musculoskelet Disord. 2013;14:348. Epub 2013/12/18. doi: 10.1186/1471-2474-14-348 ; PubMed Central PMCID: PMC4028952. 24330430 PMC4028952

[5] Seitz AL, McClure PW, Finucane S, Boardman ND 3rd, Michener LA. Mechanisms of rotator cuff tendinopathy: intrinsic, extrinsic, or both? Clinical biomechanics. 2011;26(1):1–12. Epub 2010/09/18. doi: 10.1016/j.clinbiomech.2010.08.001 . 20846766

[6] Hashimoto T, Nobuhara K, Hamada T. Pathologic evidence of degeneration as a primary cause of rotator cuff tear. Clinical orthopaedics and related research. 2003;(415):111–20. Epub 2003/11/13. doi: 10.1097/01.blo.0000092974.12414.22 . 14612637

[7] Nyffeler RW, Werner CM, Sukthankar A, Schmid MR, Gerber C. Association of a large lateral extension of the acromion with rotator cuff tears. The Journal of bone and joint surgery American volume. 2006;88(4):800–5. Epub 2006/04/06. doi: 10.2106/JBJS.D.03042 . 16595470

[8] Soslowsky LJ, Thomopoulos S, Esmail A, Flanagan CL, Iannotti JP, Williamson JD 3rd, et al. Rotator cuff tendinosis in an animal model: role of extrinsic and overuse factors. Ann Biomed Eng. 2002;30(8):1057–63. Epub 2002/11/27. doi: 10.1114/1.1509765 . 12449766

[9] Hughes RE, Bryant CR, Hall JM, Wening J, Huston LJ, Kuhn JE, et al. Glenoid inclination is associated with full-thickness rotator cuff tears. Clinical orthopaedics and related research. 2003;(407):86–91. Epub 2003/02/05. doi: 10.1097/00003086-200302000-00016 . 12567135

[10] Lee M, Chen JY, Liow MHL, Chong HC, Chang P, Lie D. Critical Shoulder Angle and Acromial Index Do Not Influence 24-Month Functional Outcome After Arthroscopic Rotator Cuff Repair. The American journal of sports medicine. 2017;45(13):2989–94. Epub 2017/08/15. doi: 10.1177/0363546517717947 . 28806093

[11] Moor BK, Wieser K, Slankamenac K, Gerber C, Bouaicha S. Relationship of individual scapular anatomy and degenerative rotator cuff tears. Journal of shoulder and elbow surgery. 2014;23(4):536–41. Epub 2014/02/01. doi: 10.1016/j.jse.2013.11.008 . 24480324

[12] Garcia GH, Liu JN, Degen RM, Johnson CC, Wong AC, Dines DM, et al. Higher critical shoulder angle increases the risk of retear after rotator cuff repair. Journal of shoulder and elbow surgery. 2017;26(2):241–5. Epub 2016/09/07. doi: 10.1016/j.jse.2016.07.009 . 27594085

[13] Li H, Chen Y, Chen J, Hua Y, Chen S. Large Critical Shoulder Angle Has Higher Risk of Tendon Retear After Arthroscopic Rotator Cuff Repair. The American journal of sports medicine. 2018;46(8):1892–900. Epub 2018/05/04. doi: 10.1177/0363546518767634 . 29723034

[14] Gerber C, Catanzaro S, Betz M, Ernstbrunner L. Arthroscopic Correction of the Critical Shoulder Angle Through Lateral Acromioplasty: A Safe Adjunct to Rotator Cuff Repair. Arthroscopy. 2018;34(3):771–80. Epub 2017/11/05. doi: 10.1016/j.arthro.2017.08.255 . 29100767

[15] Katthagen JC, Marchetti DC, Tahal DS, Turnbull TL, Millett PJ. The Effects of Arthroscopic Lateral Acromioplasty on the Critical Shoulder Angle and the Anterolateral Deltoid Origin: An Anatomic Cadaveric Study. Arthroscopy. 2016;32(4):569–75. Epub 2016/02/21. doi: 10.1016/j.arthro.2015.12.019 . 26895784

[16] Marchetti DC, Katthagen JC, Mikula JD, Montgomery SR, Tahal DS, Dahl KD, et al. Impact of Arthroscopic Lateral Acromioplasty on the Mechanical and Structural Integrity of the Lateral Deltoid Origin: A Cadaveric Study. Arthroscopy. 2017;33(3):511–7. Epub 2016/11/07. doi: 10.1016/j.arthro.2016.08.015 . 27815011

[17] Billaud A, Cruz-Ferreira E, Pesquer L, Abadie P, Carlier Y, Flurin PH. Does the critical shoulder angle decrease after anterior acromioplasty? Arch Orthop Trauma Surg. 2019;139(8):1125–32. Epub 2019/03/15. doi: 10.1007/s00402-019-03163-1 . 30868217

[18] Walch G, Edwards TB, Boulahia A, Nove-Josserand L, Neyton L, Szabo I. Arthroscopic tenotomy of the long head of the biceps in the treatment of rotator cuff tears: clinical and radiographic results of 307 cases. Journal of shoulder and elbow surgery. 2005;14(3):238–46. Epub 2005/05/13. doi: 10.1016/j.jse.2004.07.008 . 15889020

[19] Jost B, Koch PP, Gerber C. Anatomy and functional aspects of the rotator interval. Journal of shoulder and elbow surgery. 2000;9(4):336–41. Epub 2000/09/09. doi: 10.1067/mse.2000.106746 . 10979532

[20] Blonna D, Giani A, Bellato E, Mattei L, Calo M, Rossi R, et al. Predominance of the critical shoulder angle in the pathogenesis of degenerative diseases of the shoulder. Journal of shoulder and elbow surgery. 2016;25(8):1328–36. Epub 2016/02/24. doi: 10.1016/j.jse.2015.11.059 . 26899036

[21] Sankaranarayanan S, Saks BR, Holtzman AJ, Tabeayo E, Cuomo F, Gruson KI. The critical shoulder angle (CSA) in glenohumeral osteoarthritis: Does observer experience affect measurement reliability on plain radiographs? J Orthop. 2020;22:160–4. Epub 2020/05/19. doi: 10.1016/j.jor.2020.04.004 ; PubMed Central PMCID: PMC7215095. 32419757 PMC7215095

[22] Moor BK, Bouaicha S, Rothenfluh DA, Sukthankar A, Gerber C. Is there an association between the individual anatomy of the scapula and the development of rotator cuff tears or osteoarthritis of the glenohumeral joint?: A radiological study of the critical shoulder angle. Bone Joint J. 2013;95-B(7):935–41. Epub 2013/07/03. doi: 10.1302/0301-620X.95B7.31028 . 23814246

[23] Lin CL, Chen YW, Lin LF, Chen CP, Liou TH, Huang SW. Accuracy of the Critical Shoulder Angle for Predicting Rotator Cuff Tears in Patients With Nontraumatic Shoulder Pain. Orthop J Sports Med. 2020;8(5):2325967120918995. Epub 2020/06/02. doi: 10.1177/2325967120918995 ; PubMed Central PMCID: PMC7232055. 32478116 PMC7232055

[24] Neer CS 2nd. Anterior acromioplasty for the chronic impingement syndrome in the shoulder. 1972. The Journal of bone and joint surgery American volume. 2005;87(6):1399. Epub 2005/06/03. doi: 10.2106/JBJS.8706.cl . 15930554

[25] Hyvonen P, Lohi S, Jalovaara P. Open acromioplasty does not prevent the progression of an impingement syndrome to a tear. Nine-year follow-up of 96 cases. The Journal of bone and joint surgery British volume. 1998;80(5):813–6. Epub 1998/10/13. doi: 10.1302/0301-620x.80b5.8533 . 9768891

[26] Gerber C, Snedeker JG, Baumgartner D, Viehofer AF. Supraspinatus tendon load during abduction is dependent on the size of the critical shoulder angle: A biomechanical analysis. Journal of orthopaedic research: official publication of the Orthopaedic Research Society. 2014;32(7):952–7. Epub 2014/04/05. doi: 10.1002/jor.22621 . 24700399

[27] Spiegl UJ, Horan MP, Smith SW, Ho CP, Millett PJ. The critical shoulder angle is associated with rotator cuff tears and shoulder osteoarthritis and is better assessed with radiographs over MRI. Knee surgery, sports traumatology, arthroscopy: official journal of the ESSKA. 2016;24(7):2244–51. Epub 2015/03/31. doi: 10.1007/s00167-015-3587-7 . 25820655

[28] Altintas B, Kaab M, Greiner S. Arthroscopic lateral acromion resection (ALAR) optimizes rotator cuff tear relevant scapula parameters. Arch Orthop Trauma Surg. 2016;136(6):799–804. Epub 2016/02/28. doi: 10.1007/s00402-016-2431-y . 26920400

